# Supplementary material for: Application of physiological network mapping in the prediction of survival in critically ill patients with acute liver failure
Source: Sci Rep. 2024 Oct 9;14:23571. doi: 10.1038/s41598-024-74351-2 (PMC11464518; doi:10.1038/s41598-024-74351-2)
Supplement: Supplementary file 1 — Supplementary Material 1 [file 41598_2024_74351_MOESM1_ESM.docx]

**Supplementary Materials**

**Table S1.** Principal components after Varimax and rotation and Kaiser Normalization. The KMO (Kaiser-Meyer-Olkin) test that the sample is adequate for PCA showed p values < 0.001 (Chi-Square = 1844.299, p-value = <0.001).

|  | **Principal Components** | | | | | | | | |
| --- | --- | --- | --- | --- | --- | --- | --- | --- | --- |
| **Variables** | 1 | 2 | 3 | 4 | 5 | 6 | 7 | 8 | 9 |
| Alanine Aminotransferase | 0.828 |  |  |  |  |  |  |  |  |
| Aspartate Transaminase | 0.817 |  |  |  |  |  |  |  |  |
| International Normalized Ratio | 0.764 |  |  |  |  |  |  |  |  |
| Oxygen Saturation |  |  |  |  |  |  |  |  |  |
| Serum Creatinine |  | 0.845 |  |  |  |  |  |  |  |
| Urea |  | 0.807 |  |  |  |  |  |  |  |
| Phosphate |  | 0.69 |  |  |  |  |  |  |  |
| Serum Albumin |  |  | 0.692 |  |  |  |  |  |  |
| Mean Blood Pressure |  |  | 0.649 |  |  |  |  |  |  |
| Haemoglobin |  |  | 0.607 |  |  |  |  |  |  |
| Alkaline Phosphatase |  |  |  |  |  |  |  |  |  |
| Serum Sodium |  |  |  | 0.924 |  |  |  |  |  |
| Chloride |  |  |  | 0.831 |  |  |  |  |  |
| Bicarbonate |  |  |  |  | 0.801 |  |  |  |  |
| Blood pH |  |  |  |  | 0.765 |  |  |  |  |
| Heart Rate |  |  |  |  |  | 0.815 |  |  |  |
| Respiratory Rate |  |  |  |  |  | 0.707 |  |  |  |
| Blood Glucose |  |  |  |  |  |  | 0.809 |  |  |
| Lactate |  |  |  |  |  |  | 0.571 |  |  |
| Glasgow Coma Score |  |  |  |  |  |  |  |  |  |
| Platelet Count |  |  |  |  |  |  |  | 0.836 |  |
| White Blood Count |  |  |  |  |  |  |  | 0.757 |  |
| Total Bilirubin |  |  |  |  |  |  |  |  | 0.744 |
| Body Temperature |  |  |  |  |  |  |  |  | -0.728 |

*Na; Serum Sodium, Cl; chloride, AST; aspartate transaminase, ALT; alanine aminotransferase, GCS; Glasgow Coma Score, Bil; Total Bilirubin, ALP; Alkaline Phosphatase, Cr; Serum Creatinine, Na; Serum Sodium, Glu; Blood Glucose, HR; Heart Rate, Temp; Temperature, SEM; Standard Error of Mean, HR; Hazard Ratio, CI; Confident Interval.*

**Table S2.** Univariate Cox regression analysis of the Principal Components based on ICU survival of patients.

| **Principal component** | **β** | **SEM** | **Hazard Ratio (95% CI)** | **p-value** |
| --- | --- | --- | --- | --- |
| PC-1 | 0.255 | 0.092 | 1.08 (1.29 - 1.55) | **0.005** |
| PC-2 | 0.168 | 0.097 | 0.98 (1.18 - 1.43) | 0.082 |
| PC-3 | -0.258 | 0.093 | 0.64 (0.77 - 0.93) | **0.005** |
| PC-4 | 0.13 | 0.106 | 0.93 (1.14 - 1.4) | 0.222 |
| PC-5 | -0.084 | 0.102 | 0.75 (0.92 - 1.12) | 0.414 |
| PC-6 | 0.111 | 0.107 | 0.91 (1.12 - 1.38) | 0.299 |
| PC-7 | 0.226 | 0.103 | 1.03 (1.25 - 1.53) | **0.027** |
| PC-8 | -0.070 | 0.099 | 0.77 (0.93 - 1.13) | 0.478 |
| PC-9 | -0.070 | 0.099 | 0.77 (0.93 - 1.13) | 0.478 |

SEM; Standard Error of Mean, CI; Confident Interval.
